# Rheological and Film-Forming Properties of Chitosan Composites

**DOI:** 10.3390/ijms23158763

**Published:** 2022-08-06

**Authors:** Katarzyna Lewandowska, Marta Szulc

**Affiliations:** Department of Biomaterials and Cosmetic Chemistry, Faculty of Chemistry, Nicolaus Copernicus University in Toruń, Gagarin 7, 87-100 Torun, Poland

**Keywords:** chitosan, clay, rheological properties, surface properties

## Abstract

Chitosan (Chit) and its composite films are widely used in biomedical, cosmetic, and packaging applications. In addition, their properties can be improved and modified using various techniques. In this study, the effect of the type of clay in Chit composites on the structure, morphology, and physical properties of Chit solution and films was tested. The liquid flow properties of Chit solution with and without clay were carried out using the steady shear test. Chit films containing clay were obtained using the solution-casting method. The morphology, structure, and physical properties of the films were characterized by scanning electron microscopy, atomic force microscopy, infrared spectroscopy, swelling behavior, and tensile tests. The results reveal that for the Chit solution with clay (C1) containing 35 wt.% dimethyl dialkyl (C14–C18) amine, the apparent viscosity is the highest, whereas Chit solutions with other clays show reduced apparent viscosity. Rheological parameters of Chit composites were determined by the power law and Cross models, indicating shear-thinning behavior. Analytical data were compared, and show that the addition of clay is favorable to the formation of intermolecular interactions between Chit and clay, which improves in the properties of the studied composites.

## 1. Introduction

Chitosan (Chit) is a chitin derivative, commonly obtained from crustacean shells by partial deacetylation in an alkaline environment, biological process, or a combination of both processes. It is also produced by other organisms, e.g., fungi [1,2,3]. Chitin is a substance that is hard to dissolve. In contrast, Chit can be dissolved in dilute organic and inorganic acids, such as lactic acid, formic acid, acetic acid, and hydrochloric acid, which are harmless to humans and the environment. Additionally, the type of acid used significantly impacts the physicochemical properties of Chit solutions and other materials, e.g., films and hydrogels [4,5,6,7]. Chit possesses many excellent features, such as biodegradability, non-toxicity towards humans, bioactivity, biocompatibility, film-formation ability, and applicability in the preparation of biomaterials [1,8,9,10]. Thus, Chit is applied in the cosmetic, pharmaceutical, and medicine industries. However, disadvantages related to Chit as a biomaterial include rapid degradation, poor mechanical stability, and brittleness. Therefore, it is necessary to modify Chit-based materials via the addition of other substances such as ionic liquids, inorganic additives, clays, hydroxyapatites, and other polymers. Over the past two decades, many studies investigated the physicochemical properties and structure of Chit materials using different experimental techniques [7,11,12,13,14,15,16,17,18]. Reports show that the mechanical properties of Chit composites increase significantly with increasing concentrations of clays [19,20,21]. Infrared spectroscopy analysis and thermal properties prove that interactions occur between Chit matrix and clays [21,22,23,24]. Hence, mixing with clay is a promising process to physically modify Chit, providing enhanced properties for various applications. In this study, we used three different types of clays to modify Chit materials for potential biomedical and cosmetic applications. Clays are layered hydrated alumina–silica. In addition, surfaces of layered silicate can be modified with alkylammonium surfactants, quaternary ammonium salts, or organosilicon compounds to produce organoclays [16,20,25,26]. Therefore, Chit and clay composites are considered as interesting hybrid materials for potential application in medicine.

Herein, we determined the effect of the addition of montmorillonite clay on the properties of the composite in the formation of thin films. Thin films were formed using the casting method from lactic acid solution. Our study is a progressive continuation of the characterization of unique Chit composites [27,28,29,30]. In the presented research, we used two clay types and a variety of methods i.e., shear steady tests and swelling test, as well as using lactic acid as solvent. Lactic acid is utilized in pharmaceutical and cosmetic formulations for the production of hygiene and esthetic products, owing to its moisturizing, antimicrobial, and rejuvenating effects on the skin. Additionally, it is employed in oral hygiene products, and supplemented in the synthesis of dermatologic drugs and against osteoporosis [31]. Information about the rheological and film-forming of these modified Chit solutions and films is essential from a practical point of view, as it allows for the estimation of the possibilities for cosmetic and pharmaceutical usage. Based on rheological research, one could design materials with tailor-made properties for unique applications, e.g., tissue engineering purposes. In addition, a new aspect of this research was to compare the morphology and properties of the composite films before and after alkaline treatment. Analyses of morphology, surface properties, and swelling behavior, as well as mechanical tests, provided important information. For many applications, such as tissue engineering, drug delivery, or wound healing, it is crucial to know the swelling behavior in physiological solution, which is usually simulated by phosphate-buffered saline (PBS). Swelling behavior is a key feature of Chit materials, and characterizes its applications for biomedical application, especially tissue engineering and material construct.

## 2. Results and Discussion

### 2.1. Steady Shear Rheological Studies

**Figure 1:**
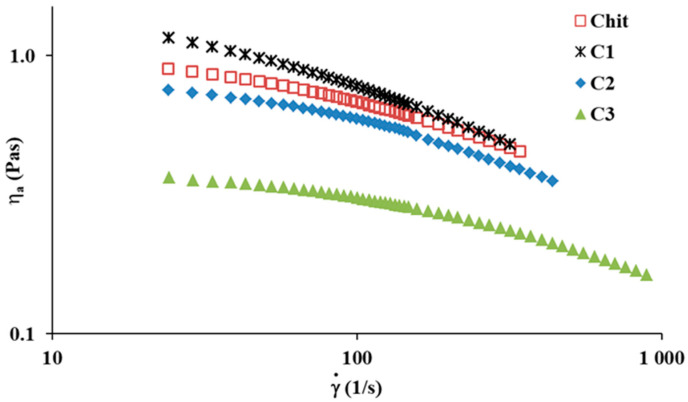
Curves of apparent viscosity vs. shear rate for Chit solution and Chit/clay mixtures at 25 °C.

The steady shear tests determined the apparent viscosity (*η_a_*), the rheological parameters from the Ostwald–de Waele model (Equation (1)) and Cross model (Equation (2)), and the activation energy of viscous flow (Equation (3)) for the Chit solution and Chit/clay mixtures. Figure 1 shows the apparent viscosity curves as a function of the used shear rate for Chit solutions, with and without clay, at 25 °C.

All investigated solutions display shear-thinning behavior over a wide range of shear rates. This is in accordance with previous reports [13,32,33,34], where a decrease in apparent viscosity with the shear rate stems from the disentanglement of macromolecules, induced by shear forces, and dispersed polymer molecules arrange along the flow direction [16,34]. In the case of the C1 mixture solution, the apparent viscosity is higher than that of the pure Chit solution, especially for a lower shear rate (<100 s^−1^). The addition of C2 or C3 to the Chit solution causes a reduction in apparent viscosity. These observations are attributed to the different types of interactions, including repulsive forces, hydrophobic interaction, and van der Waals forces, as well as hydrogen bonds between Chit molecules and clays and ion pairs with solvent (i.e., CH_3_CH(OH)COO^−^/H_3_O^+^) in the mixture, resulting in increased viscosity values for C1, and decreased viscosity for both C2 and C3. Interactions must exist between both components of the mixture, in addition to the interactions mentioned above, such as interactions between each component and solvent, and between segments of Chit. Therefore, all such interactions contribute to the change in apparent viscosity.

The rheological parameters of Chit solutions with and without clay obtained by fitting the experimental data to the different rheological models are presented in Table 1.

The correlated coefficients are all larger than 0.99, demonstrating that the studied Chit solutions fit the examined models. Based on the classical power law model, all Chit solutions exhibit shear-thinning behavior, characterized by the non-Newtonian index (*n*) values in the range of 0.64 to 0.76. C1 exhibits the highest *k* and lowest *n* values in the studied solutions. The obtained rheological parameters (*n* and *k*) from the power law model suggest a high association degree, and complex formation, between components in C1. In the case of Chit/clay mixtures, the Chit chains are stretched due to repulsive forces between positive charges on the amine groups, which increases the solution’s viscosity. Thus, temporary networks or entanglements of the unfolded macromolecules could possibly form under flow conditions. The shear force may disturb the screening of electrostatic charges by opposite ions (from the solvent) and, consequently, the repulsive force between positive charges in Chit/C1 mixture solutions increases *k* parameters. The consistency index (*k*) value of the Chit solution significantly decreases after the addition of C3 (Table 1), suggesting that the addition of C3 results in less flow resistance. This effect may be due to the lack of interactions between Chit chains, and weaker repulsive force between the Chit molecules and C3. Hence, the apparent viscosity and consistency index decreases significantly compared to other composite solutions. This study also employed the Cross model to describe the viscosity profile of Chit solutions over a wide shear rate range, because of the limitations of the power law model in determining the zero-shear and infinite viscosities. The limiting Newtonian viscosity at low shear rate (*η*_0_) and high shear rate (*η*_∞_) appears in the range of 1.59–0.40 Pas and 0.197–0.0497 Pas, respectively. Generally, the zero-shear viscosity is mainly associated with the number of interactions between the molecules in fluid state, where higher values suggest a higher number of interactions [35]. Thus, an increase in *η*_0_ from 1.08 (Chit) to 1.59 Pas (C1) suggests the occurrence of stronger intermolecular interactions between components, which are responsible for the viscosity increase after C1 addition. In the case of the addition of C2 and C3, this variation is attributed to the structure of the Chit solution becoming looser and inter/intra-molecular friction forces decreasing, leading to reduced viscosity prior to shear measurements.

The influence of temperature on the apparent viscosity for the Chit solution and its composites was studied. Table 2 shows the activation energy changes in the viscous flow of Chit solutions with and without clay. In general, *E_a_* values decrease with increasing shear rate, and after the addition of clay to the Chit solution. *E_a_* values are lower than the pure Chit solution. The lowest *E_a_* values are found for C1. The results show that the viscosity of the mixture solutions exhibit the lowest sensitivity to temperature, due to the addition of clay-inducing assembly among active groups on Chit molecules strengthening the network structure.

### 2.2. Mechanical Studies

**Figure 2:**
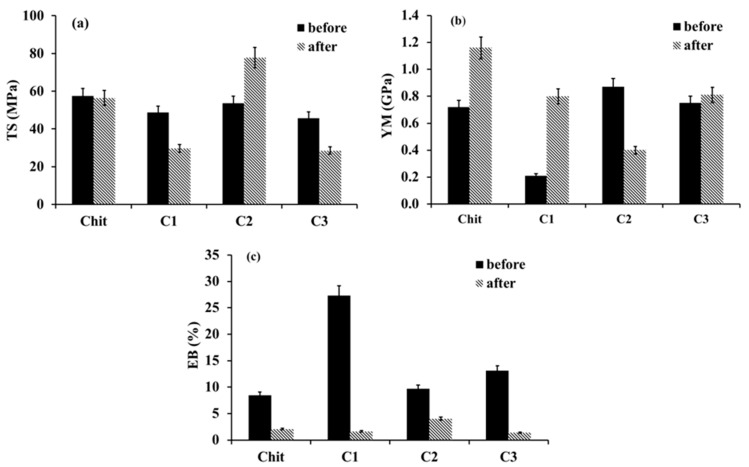
Mechanical properties of Chit and Chit composite films: (**a**) TS, (**b**) YM, and (**c**) EB, before and after alkaline treatment, *n* = 5, mean ± SD (standard deviation), error bars represent SD.

The influence of clay addition to Chit composites before and after alkaline treatment on the mechanical properties, such as tensile strength (TS), Young’s modulus (YM), and percentage elongation at the break (EB), were studied. Figure 2 shows the mechanical properties of Chit and composite films before and after neutralization.

It is observed that Chit composite films before alkaline treatment exhibit YM values ranging from 0.21 GPa ± 0.02 to 0.87 GPa ± 0.06 (Figure 2) compared to Chit films (0.72 GPa ± 0.05). The minimum YM value (0.21 GPa ± 0.02) is obtained for C1 composite films. TS values of composite films range from 45.70 MPa ± 3.99 to 53.60 MPa ± 3.75, and are similar to those for pure Chit films (57.42 Mpa ± 4.10). For Chit film, the EB value is 8.50% ± 0.60. After the addition of clay to Chit solution, all films show increased EB values, from 9.7% ± 0.6 to 27.30% ± 1.90. The maximum EB value (27.30% ± 1.90) is achieved by the addition of C1. This can be explained by the interactions between Chit molecules and clays, as well as solvent. As shown in rheological studies, the addition of clays to the Chit solution leads to a reduction of intramolecular hydrogen bonds between polymer chains, and creates new intermolecular interactions between components. These interactions alter the polymer networks, and lead to an improvement in the flexibility of the polymer films. Hence, Chit composite films could allow for the preparation of materials with improved elastic properties. After alkaline treatment, all films show reduced EB values. The minimum value of EB is achieved by the C1 (1.66% ± 0.12) and C3 composite films (1.40% ± 0.10). The Chit film possesses an EB value of 2.60% ± 0.18. The observed changes in the elastic properties of the films are related to the removal of residual lactic acid, and the intermolecular interactions between the polymer matrix and clays. Additionally, the neutralization leads to changes in the arrangement and packing of polymer chains, and, thus, to a change in the surface topography and physical properties of Chit films. Reports show that some carboxylic acid decreases TS and YM while increasing EB of the Chit film, indicating that some unreacted acid acts as a plasticizing agent [36,37,38]. However, YM values increase compared to the same type of films before neutralization, with the exception of C2 (0.40 GPa ± 0.04). In the case of the Chit film, YM is 1.16 GPa ± 0.08, whereas C1 and C3 are 0.80 GPa ± 0.06 and 0.83 GPa ± 0.07, respectively. Thus, the difference in the C3 composite film is not significant. The composite films exhibit a large YM because of their brittleness. As a result, the incorporation of clay into the polymer composite films serves as reinforcement, and leads to improved YM. Reports show that increased modulus values for composite films are mainly due to the interactions between the polymer matrix and silicate layers via the formation of hydrogen bonds and Van der Waals forces, as well as hydrophobic interactions, which produce stable structures [19,22,27,28,39].

### 2.3. Swelling Behavior

**Figure 3:**
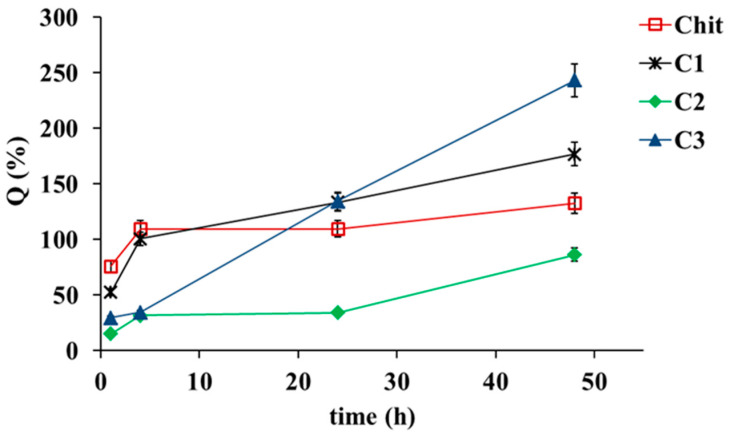
Swelling ratio of Chit films and its composite films, *n* = 3, mean ± SD (standard deviation), error bars represent SD.

The swelling properties of Chit and its composite films after neutralization were studied by measuring their weights after immersion in PBS solution (pH = 7.4) at 37 °C. The swelling curves of films after alkaline treatment are shown in Figure 3.

It is well-known that the following parameters affect the dissolution and swelling behavior of polymer films: the chemical composition of films, nature of polymer, solvent, flexibility of polymer chain, molecular mass of polymer, and chemical crosslinks [40,41,42]. According to our study, swelling properties of Chit-films-based composites are improved after the addition of C1 and C3. After 24 h of immersion, no significant differences are observed in the swelling behavior between Chit films and composite films with C1 and C3. Among the films, the highest swelling is observed for that containing C3, after 48 h of immersion. However, composite films containing C2 show a lower swelling ratio than other composite films. This observation indicates that the structure of the C2 composite film is more rigid and compact compared to other composites. The swelling ratio of Chit composite films may be related to both ionic repulsion between partial protonated groups, the formation of hydrogen bonds between Chit and silicate layers, and other types of interactions. Higher swelling ratio values are due to the presence of unbound polar groups in films, which interact with water molecules. Previous reports show that during the initial step of the hydration process, bond cleavage and degradation of Chit materials also occur, but the swelling behavior exceeds that of degradation [40,42]. In the second step, the swelling ratio reaches its maximum value, and continual degradation leads to weight loss. The polymer materials tend to be thinner, and the swelling ratio decreases [40,41,42]. Thus, the swelling studies may be useful when designing Chit-based tissue scaffold, cartilage, and drug delivery systems.

In contrast, all Chit films before alkaline treatment quickly absorb water, and dissolve in PSB solution after 1 h of immersion, which indicates that lactic acid does not form any cross-linking in the polymer matrix [41]. These results reveal that the incorporation of the proper clay into the Chit matrix improves its water absorption capacity.

### 2.4. Morphological Properties

**Figure 4:**
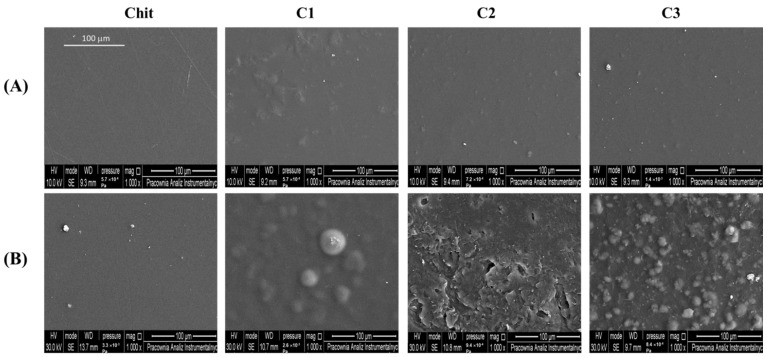
Representative SEM images of film surface of different composition (**A**) before and (**B**) after NaOH treatment (magnification ×1000, bar = 100 μm).

**Figure 5:**
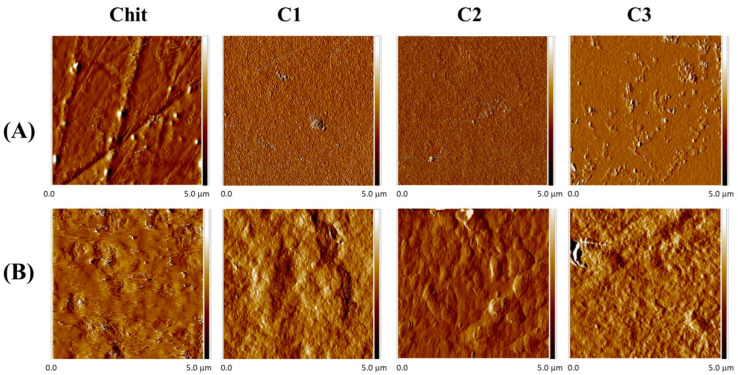
Representative AFM images of film surface of different composition (**A**) before and (**B**) after NaOH treatment.

The surface morphologies of films before and after neutralization were evaluated using the energy dispersive X-ray spectrometer (SEM–EDX) and AFM methods. EDX analysis was conducted to assess the elemental composition of Chit films. SEM and AFM images of all films are shown in Figure 4 and Figure 5. Roughness parameters of films are tabulated in Table 3.

We observe that before the neutralization process, pure Chit and composite films with C2 and C3 show a homogenous structure, with relatively smooth surface morphology. The roughness parameters of the composite films are the lowest among the studied films. However, the surface of the C1 composite film exhibits small particles, corresponding to the presence of clay 1 particles, which are homogeneously spread over the entire surface. In addition, EDX analysis (graphs not shown) exhibits the presence of magnesium (Mg), sodium (Na), aluminum (Al), and silicon (Si) elements in the composite films, thereby proving the presence of clay in the composite films.

After NaOH treatment (Figure 4 and Figure 5), SEM and AFM images show an increasing number and size of domains on the film’s surface as lactic acid is removed. The films surfaces becomes rougher after neutralization, hence, the roughness parameters increase significantly (Table 3). These observations are consistent with previous studies on Chit films [43,44]. Therefore, the compact structure of Chit films are found in hydroxylated acid films such as lactic acid, citric acid, and malic acid. This behavior is explained by the intermolecular interactions between Chit molecules and hydroxylated acid counter anions [37,45,46,47]. The aforementioned interactions affect the structure and mechanical properties of Chit films, resulting in the films formed in different conditions possessing different surface morphologies.

### 2.5. Infrared Spectroscopy

**Figure 6:**
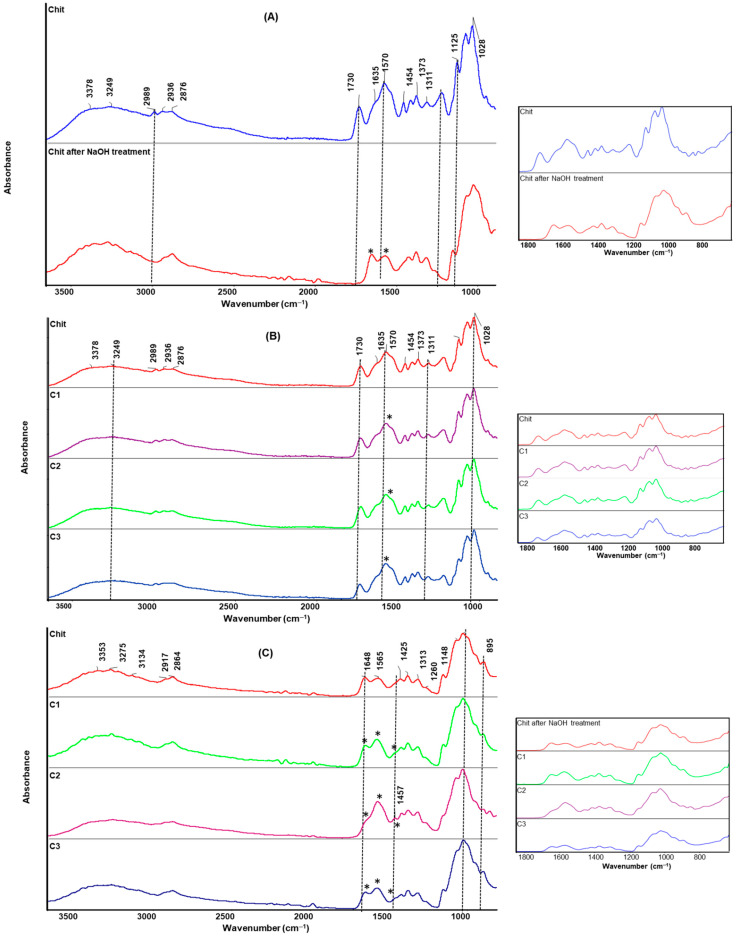
ATR infrared spectra of (**A**) Chit and Chit composite films (**B**) before and (**C**) after NaOH treatment. The inserts are magnification, showing the region of 600–1800 cm^−1^, asterisks (*) indicate changes in the characteristic bands.

Figure 6 shows IR spectra of pure Chit and Chit composite films before and after alkaline treatment. Chit films cast from lactic acid exhibit a wide band at 3249 cm^−1^, which corresponds to O–H and N–H stretching vibrations, as well as hydrogen bonds between Chit molecules [38,44,48]. The bands at 2989, 2936, and 2876 cm^−1^ are attributed to the C–H stretching vibration of methylene. Distinctive bands at 1635, 1570, 1450, and 1373 cm^−1^ correspond to the carbonyl stretching (amide I), –NH bending in amide groups (amide II), and C–N stretching vibrations (amide III), respectively [38,41,45]. The vibrations of C–O, C–O–C glycosidic. and C–O–H bands appear at 1073 and 1028 cm^−1^. The bands at 1125, 1220, and 1730 cm^−1^ are characteristic bands of lactic acid, which reveal the presence of lactic acid in all Chit films before NaOH treatment (Figure 6A,B) [38,45]. After alkaline treatment, the carbonyl absorption peaks at 1125, 1220, and 1730 cm^−1^ disappear (Figure 6A,C), which indicates that the remaining lactic acid is removed. Compared to Chit film before alkaline treatment, the intensity and shape of the amide I and II bands are changed (Figure 6A, marked with an asterisk), due to the modification of the amine group [38,48,49]. Additionally, the intensity of amide I is more significant than the amide II band. This modification reduces the hydration shell of the amine group, and allows new interactions to form in the polymer chains.

In the case of composite films, the spectra show the main characteristic bands of Chit and clay, confirming the formation of the composite films. The additional bands related to clay are located at 3500 cm^−1^ (−OH stretching vibration), and 1100 and 1020 cm^−1^ (Si–O–Si bands) [22,23]. The spectra show a noticeable change in the intensity and shape of the amide bands between 1300 and 1650 cm^−1^, and bands in the region of 1200–850 cm^−1^, which indicates that some of the amine and hydroxyl groups of Chit and hydroxylated silicate edge groups generate new interactions. The small shifts in the maximum characteristic bands in the composite films are also observed. In addition, a new, small peak at approx. 1457 cm^−1^ is observed for composite films after alkaline treatment (Figure 6C, marked with an asterisk). This may indicate that the interactions between the Chit and silicate layers via the formation of hydrogen bonds and Van der Waals forces, as well as hydrophobic interactions. The obtained FTIR results are in agreement with the rheological and mechanical tests, which indicate that new interactions between Chit matrix and silicate layers modify its apparent viscosity and physical properties.

## 3. Materials and Methods

### 3.1. Materials

Chit powder with 80% degree of deacetylation (DD) and viscosity average molecular weight of 1382 kg/mol was purchased from Marine Fisheries Research Institute (Gdynia, Poland). Viscosity average molecular weight was obtained using the Mark–Houwink–Sakurada equation [50]. The intrinsic viscosity was determined by viscometric technique, using a Ubbelohde viscometer. Chit in 0.1 mol/L acetic acid/0.2 mol/L NaCl exhibited the following constants: K = 1.81 × 10^−3^ mL/g and a = 0.93 at 25 °C [51]. DD of Chit was estimated by potentiometric titration [52,53]. To create Chit composites, three different types of montmorillonite clays were used: (1) surface-modified containing 35 wt.% dimethyl dialkyl (C14-C18) amine, (2) surface-modified containing 25 wt.% trimethyl stearyl ammonium, and (3) surface-modified containing 25 wt.% methyl dihydroxyethyl hydrogenated tallow ammonium. The aforementioned clays were purchased from Sigma-Aldrich (Poznań, Poland). All other chemicals utilized in this research were purchased from Chempur (Piekary Śląskie, Poland) and POCh (Avantor, Gliwice, Poland). Materials were of analytical grade, and used without any further purification.

### 3.2. Solutions and Films Preparation

Chit was dissolved in 0.3 mol/L lactic acid at 2% concentration. Clay powder (3% relative to Chit) was dispersed in 5 mL of water and stirred for 1 h. Then, the Chit solution was slowly added to the clay dispersion, and the resulting composite was vigorously stirred using a magnetic stirrer for 24 h at room temperature. The prepared Chit solution was mixed separately with three clays, including clay 1, clay 2, and clay 3, forming composites C1, C2, and C3, respectively. These solutions were used to study liquid flow properties. The solutions were also placed into squared, plastic Petri dishes (100 × 15 mm), and dried at room temperature for 72 h. Then, the prepared films were peeled off and examined. Each type of film was neutralized through immersion in 1% sodium hydroxide (NaOH) solution for 15 min. The films were rinsed and placed in distilled water overnight. After air-drying, the films were stored in a desiccator prior to mechanical tests, swelling behavior determination, and SEM and infrared analyses.

### 3.3. Steady Shear Tests

Flow measurements of Chit solution and its mixture solutions were performed on a Bohlin Visco BV 88 rotary viscometer with concentric cylinder (Marlvern, Panalytical, Malvern, UK), at various temperatures (25–40 °C) and shear rates (20–1230 s^−1^). Power law model (Equation (1)) and Cross model (Equation (2)) were adopted to fit the viscosity curves, which properly described the relationship between apparent viscosity and shear rate in chitosan mixtures [54,55]:

(1) $$\eta_{a}=k{\dot{\gamma }}^{n-1},$$

where *η_a_* is apparent viscosity, *k* is consistency index (Pas)*^n^*, $\dot{\gamma }$ is shear rate (1/s), and *n* is non-Newtonian index (dimensionless). For non-Newtonian liquids, the rheological behavior was described as a shear-thinning behavior when *n* < 1, and as a shear-thickening behavior when *n* > 1.

(2) $$\eta_{a}=\eta_{\infty }\frac{\eta_{0}-\eta_{\infty }}{1+\lambda {\dot{\gamma }}^{m}},$$

where $\eta_{0}$ is zero-shear viscosity (Pas), $\eta_{\infty }$ is infinite viscosity (Pas), *λ* is characteristic relaxation time (s), and m is constant (dimensionless).

The impact of temperature on apparent viscosity of the mixture solutions was obtained according to Arrhenius equation (Equation (3)) [35,56]:

(3) $$\eta_{0}=Aexp\left(\frac{E_{a}}{RT}\right),$$

where *E_a_* is activation energy of viscous flow (kJ mol^−1^), *R* is gas constant (8.314 kJ mol^−1^ K^−1^), and *T* is absolute temperature (K). The activation energy of viscous flow (*E_a_*) was obtained from the slope of ln *η*_0_ vs. 1/T curve.

### 3.4. Mechanical Test

Tensile strength (TS), Young’s modulus (YM), and percentage elongation at the break (EB) were measured using a Z05 Zwick Roell (Zwick Roell, Ulm, Germany) at a crosshead speed of 50 mm/min, in accordance with standard procedure under dry conditions at room temperature [57]. The tested samples were cut using the same shaper. The size of the sample was 10 mm in width and 25 mm in parallel length. A total of 5 samples of each type of film were tested.

### 3.5. Swelling Behavior

The swelling behavior of Chit composite films was determined at 37 °C in phosphate-buffered saline (PBS). After drying in a vacuum oven at 45 °C for 48 h, the films with a size of 1 × 1 cm^2^ were immersed in PBS at pH = 7.4 and 37 °C [15,58]. After the incubation time 1, 4, 24, and 48 h, the films were gently dried by placing them between two sheets of paper and weighed. The swelling ratio of films (Q) was calculated using Equation (4):

(4) $$Q=\frac{\left(m_{w}-m_{d}\right)}{m_{d}}*100\%,$$

where *m_w_* is weight of wet film, and *m_d_* is weight of dry film. Three samples of each type of film were tested, and the mean values were taken as swelling ratio.

### 3.6. Scanning Electron Microscopy (SEM)

The surface morphology of the composite films and the elemental composition of the polymer samples before and after the neutralization process were examined using a scanning electron microscope (SEM), (LEO 1430 VP model, Electron Microscopy Ltd., Cambridge, England) coupled with an energy dispersive X-ray spectrometer (EDX) (Quantax 200 with XFlash 4010 Detector, Bruker, AXS, Berlin, Germany).

### 3.7. Atomic Force Microscopy (AFM)

Surface imaging was also analyzed using an atomic force microscope (AFM) (Multimode Scanning Probe Microscope Nanoscope IIIa, Digital Instruments, Veeco Metrology Group, Santa Barbara, CA, USA), operating in tapping mode at room temperature under an air atmosphere. The root-mean-square (*R_q_*) roughness values were calculated from a 5 μm × 5 μm scanned area using NanoScope Analysis v1.40 software (Bruker, Ettlingen, Germany).

### 3.8. Infrared Spectroscopy (ATR-FTIR)

Spectra of each type of composite films were recorded using a Vertex 70v FT-IR spectrophotometer (Bruker Optics Inc., Billerica, MA, USA), in attenuated total reflectance (ATR) node with a diamond crystal in the range of 4000–600 cm^−1^ over 64 scans, and at a resolution of 2 cm^−1^.

## 4. Conclusions

Chit/clay composites before and after NaOH treatment were prepared and characterized. According to the obtained viscosity plots, the Chit and Chit/clay mixture solutions were determined as typical shear-thinning fluids. The addition of clay 1 to Chit solutions increases its apparent viscosity. The flow behavior of the studied solutions was well described using the power law and Cross models. The non-Newtonian index of all solutions is below one, indicating shear-thinning behavior. Furthermore, the results show that the addition of clay and alkaline treatment also improves the impact on the film’s properties. Chit is found to interact with lactic acid and clay through various types of interactions, including repulsive forces, hydrophobic interaction, van der Waals forces, and hydrogen bonding, as well as the modification of the properties of films based on such composites. The addition of clay to Chit film improves its elasticity. Alkaline treatment promotes pronounced surface changes, and increases roughness of the relatively smooth surface of the composite films. The largest increase is observed for C1 composite films. Chit films neutralized with NaOH exhibit higher stiffness than those before neutralization. The swelling properties of Chit films show a substantial increase after the addition of clay 1 and 3. The results of the study are helpful for the preparation of Chit materials based on Chit/clay composites for different applications, such as tissue engineering, wound healing, and cosmetics, especially hair care.

## Figures and Tables

**Table 1:** Rheological parameters obtained from power law model and Cross model for Chit solution and Chit/clay mixtures at 25 °C.

Power Law Model				Cross Model				
Sample	*n*	*k* (Pas)^n^	*R* ^2^	*η*_0_ (Pas)	*η*_∞_ (Pas)	*λ* (1/s)	*m*	*R* ^2^
Chit	0.73	2.35	0.999	1.08	0.0863	0.0196	0.77	1.00
C1	0.64	3.93	0.998	1.59	0.0878	0.0377	0.75	1.00
C2	0.72	2.07	0.997	0.80	0.197	2.78 × 10^−3^	1.14	1.00
C3	0.76	0.89	0.998	0.40	0.0497	7.79 × 10^−3^	0.82	1.00

**Table 2:** *E_a_* values of Chit solution and Chit/clay mixtures at various shear rates.

Sample	*E_a_* (kJ/mol)			
	0 s^−1^	R^2^	247 s^−1^	R^2^
Chit	38.62	0.993	33.39	0.995
C1	32.92	0.969	25.27	1
C2	35.21	0.997	30.49	1
C3	37.13	0.993	29.36	1

**Table 3:** Roughness parameters of films with various compositions before and after NaOH treatment.

Sample	Before	After
	*R*_q_ (nm)	*R*_q_ (nm)
Chit	3.75	6.77
C1	1.07	64.4
C2	0.529	47.0
C3	3.08	32.6

## Data Availability

Data are contained within the article.
